# Supplementary material for: Multi-AGV path planning method for the textile industry based on improved ant colony optimization algorithm and R-tree line-segment bilayer conflict detection
Source: PLoS One. 2025 Dec 30;20(12):e0339583. doi: 10.1371/journal.pone.0339583 (PMC12753090; doi:10.1371/journal.pone.0339583)
Supplement: Attachment — Submitted filename: Response to Reviewers.docx [file pone.0339583.s005.docx]

Dear Editor and Reviewers,

Thank you for offering us an opportunity to improve the quality of our submitted manuscript (Article Number: [PONE-D-25-27260] / Title: “[Multi-AGV path planning method for the textile industry based on improved ant colony optimization algorithm and R-tree line-segment bilayer conflict detection]”). We appreciated very much the reviewers’ constructive and insightful comments. In this revision, we have addressed all of these comments/suggestions. We hope the revised manuscript has now met the publication standard of your journal.

We have highlighted all the revisions in red. Below we provide point-by-point responses and indicate where the corresponding revisions were made.

**Reviewer #1:**

**(1) In the RLBCD model, there are numerous line segments. How do you determine the length of each line segment? What is the basis for dividing the lines into segments? Please provide more detailed information on this aspect.**

**Response:**

Thank you for asking for a crisper definition—this question goes to the heart of RLBCD’s correctness and we appreciate your precision. We now make the segmentation rule explicit in RLBCD model: after IACO, each AGV path is partitioned into co-directional segments using turning-coordinate indices; then each segment is subdivided by consecutive node pairs with well-defined time windows. Coarse screening uses (i) time-window overlap and (ii) segment-to-segment minimum distance vs.$D_{\text{safe}}$; fine screening verifies on subdivided segment units. See Eqs. (13)-(16) (definitions) and Eqs. (17)-(19) (coarse/fine checks). The specific revisions are as follows:

Upon completion of the IACO simulation, the initial path nodes and their timestamps for each AGV in the same batch can be obtained. Assume there are $n$ AGVs in this batch. For the $\alpha$-th AGV ($\alpha=1,2,...n$), let $N^{\alpha}$ denote the number of path nodes and $N_{\text{turns}}^{\alpha}$ the number of turning nodes; then the path-node set of AGV$\alpha$ is defined as $\mathcal{D}_{0}^{\alpha}$.

$$\mathcal{D}_{0}^{\alpha}=\{n_{1}^{\alpha},\ldots,n_{N^{\alpha}}^{\alpha}\},n_{1}^{\alpha}=\left( x_{1}^{\alpha},y_{1}^{\alpha},t_{1}^{\alpha} \right)$$

(13)

Based on the turning-coordinate indices, each AGV’s path can be partitioned into same-direction segments in ascending order of departure time, yielding the start/end coordinates and the traversed time windows. The set of same-direction segmented paths for AGV$\alpha$ is denoted by $\mathcal{D}_{1}^{\alpha}$ as follows:

$$\mathcal{D}_{1}^{\alpha}=\{L_{1}^{\alpha},L_{2}^{\alpha},\ldots,L_{N_{\text{turns}}^{\alpha}+1}^{\alpha}\}, L_{j}^{\alpha}=(P_{s,j}^{\alpha},P_{e,j}^{\alpha},t_{j}^{\alpha})$$

(14)

- $L_{j}^{\alpha}$: the $j$-th contiguous same-direction path segment of AGV$\alpha$

- $P_{s,j}^{\alpha}=\left( x_{s,j}^{\alpha},y_{s,j}^{\alpha} \right), P_{e,j}^{\alpha}=\left( x_{e,j}^{\alpha},y_{e,j}^{\alpha} \right)$: start and end coordinates of path segment $L_{j}^{\alpha}$

- $t_{j}^{\alpha}=(t_{s,j}^{\alpha},t_{e,j}^{\alpha}]$: line segment $L_{j}^{\alpha}$ time domain (s)

- $t_{e,j}^{\alpha}=t_{s,j+1}^{\alpha}$, $P_{e,j}^{\alpha}=P_{s,j+1}^{\alpha}$: ensure time continuity and accurate path segmentation

Define the global path-node set $\mathcal{D}_{0}$ and the global same-direction segmented path set $\mathcal{D}_{1}$ for all AGVs whose routes have been confirmed. For the AGVs departing in the current batch, if no AGV is already operating on the grid map, then $\mathcal{D}_{0}$ and $\mathcal{D}_{1}$ are empty; if some AGVs are already executing tasks, their paths are placed into $\mathcal{D}_{0}$ and $\mathcal{D}_{1}$. By the priority definition, AGVs currently executing tasks have higher priority than those departing now, and because path planning proceeds in descending priority order, the global sets $\mathcal{D}_{0}$ and $\mathcal{D}_{1}$ can be viewed as the node and path sets of higher-priority AGVs, which constitute the final outputs of the algorithm. If no AGV is executing a prior task, then upon completion of the current batch simulation the following sets are obtained.

$$\mathcal{D}_{0}=\{\mathcal{D}_{0}^{1},\ldots\mathcal{D}_{0}^{\alpha}\ldots,\mathcal{D}_{0}^{n}\}, \mathcal{D}_{1}=\{\mathcal{D}_{1}^{1},\ldots\mathcal{D}_{1}^{\alpha}\ldots,\mathcal{D}_{1}^{n}\}$$

(15)

Further, suppose the $j$-th same-direction path segment $L_{j}^{\alpha}$ is partitioned into $g_{j}^{\alpha}$ elementary line segments by connecting consecutive node pairs. Using the start/end coordinates of $L_{j}^{\alpha}$, the relevant node information can be queried from the set $\mathcal{D}_{0}^{\alpha}$. We then define the set of subdivided segments for the same-direction path $L_{j}^{\alpha}$ of AGV$\alpha$ as follows ($i$=1,2, …, $g_{j}^{\alpha}$):

$$\mathcal{D}_{2}^{\alpha}\left( L_{j}^{\alpha} \right)=\{l_{j,1}^{\alpha},l_{j,2}^{\alpha},\ldots,l_{j,g_{j}^{\alpha}}^{\alpha}\}, l_{j,i}^{\alpha}=\left( P_{s,j,i}^{\alpha},P_{e,j,i}^{\alpha},t_{j,i}^{\alpha} \right)$$

(16)

- $l_{j,i}^{\alpha}$: the $i$-th subdivided segment of the$j$-th contiguous same-direction path segment of AGV$\alpha$

- $P_{s,j,i}^{\alpha}=\left( x_{s,j,i}^{\alpha},y_{s,j,i}^{\alpha} \right), P_{e,j,i}^{\alpha}=\left( x_{e,j,i}^{\alpha},y_{e,j,i}^{\alpha} \right)$: the start and end coordinates of the subdivided segment $l_{j,i}^{\alpha}$

- $t_{j,i}^{\alpha}=(t_{s,j,i}^{\alpha},t_{e,j,i}^{\alpha}]$: the time interval of the subdivided segment $l_{j,i}^{\alpha}$ (s)

-$t_{e,j,i}^{\alpha}=t_{s,j,i+1}^{\alpha}$, $P_{e,j,i}^{\alpha}=P_{s,j,i+1}^{\alpha}$: ensure time continuity and accurate path segmentation

**RLBCD coarse screening stage**

In the coarse-screening stage, spatiotemporal conflict detection for the current-priority AGVs adopts a binary criterion: (i) whether the time windows overlap, and (ii) whether the minimum distance between segments is below the safety threshold. Segments are examined in the order of the departure times of the current-priority AGV$\alpha$: segments of $\alpha$ are taken from $\mathcal{D}_{1}^{\alpha}$, while higher-priority segments are taken from the global set $\mathcal{D}_{1}$. Let $L_{j}^{\alpha}$ be the segment currently under consideration for AGV$\alpha$, and $L_{k}^{\beta}$ the segment of a higher-priority AGV$\beta$. The candidate conflict set for $L_{j}^{\alpha}$ is defined as:

$$\mathcal{C}_{1}^{\alpha}(L_{j}^{\alpha})=\{L_{k}^{\beta}\in\mathcal{D}_{1}|t_{j}^{\alpha}\cap t_{k}^{\beta}\neq\emptyset\wedge d_{\text{min}}(L_{j}^{\alpha},L_{k}^{\beta})<D_{\text{safe}}\}$$

(17)

- $D_{\text{safe}}$: safe distance thresholds (m)

- $d_{\text{min}}$: minimum distance between line segments$L_{j}^{\alpha},L_{k}^{\beta}$ (m)

-$t_{j}^{\alpha}\cap t_{k}^{\beta}$: means the direction continuous line segment$L_{j}^{\alpha},L_{k}^{\beta}$time domain overlap

-$\emptyset$: empty set

In the coarse-screening stage, we first filter by whether the time windows overlap and then compute exact segment-to-segment distances to obtain $\mathcal{C}_{1}^{\alpha}(L_{j}^{\alpha})$. If $\mathcal{C}_{1}^{\alpha}(L_{j}^{\alpha})=\emptyset$, then $L_{j}^{\alpha}$ is deemed conflict-free and we proceed to the next contiguous segment $L_{j+1}^{\alpha}$; otherwise, the procedure enters the fine-screening stage.

**RLBCD fine screening stage**

Based on $\mathcal{C}_{1}^{\alpha}(L_{j}^{\alpha})$ and $L_{j}^{\alpha}$, retrieve the sets $\mathcal{D}_{2}^{\beta}(L_{k}^{\beta})$ and $\mathcal{D}_{2}^{\alpha}(L_{j}^{\alpha})$, and perform fine-screening for precise conflict determination. The fine-screening proceeds as follows: first, use time-window overlap to quickly filter subdivided segment units $l_{k,i^{'}}^{\beta}$ and $l_{j,i}^{\alpha}$; suppose the selected pair is $l_{j,1}^{\alpha}$ and $l_{k,2}^{\beta}$. Finally, compute the exact distance between the subdivided segments $l_{j,1}^{\alpha}$ and $l_{k,2}^{\beta}$. A conflict is confirmed if and only if the following conditions are satisfied:

$$\text{Real}\text{\_}\text{c}\text{onflict}\text{(}l_{j,1}^{\alpha},l_{k,2}^{\beta}\text{)}=\left( d_{\text{min}}(l_{j,1}^{\alpha},l_{k,2}^{\beta})<D_{\text{safe}} \right)\cap\left( t_{j,1}^{\alpha}\cap t_{k,2}^{\beta}\neq\emptyset\right)$$

(18)

After fine screening, the precise conflict-information set $\mathcal{C}_{2}^{\alpha}(L_{j}^{\alpha})$ is obtained:

$$\mathcal{C}_{2}^{\alpha}(L_{j}^{\alpha})=\left\{ (l_{j,1}^{\alpha},l_{k,2}^{\beta}),(l_{j,2}^{\alpha},l_{m,1}^{\gamma}),.... \right\}$$

(19)

- $l_{j,1}^{\alpha}=(P_{s,j,1}^{\alpha},P_{e,j,1}^{\alpha},t_{j,1}^{\alpha})$

- $(l_{j,1}^{\alpha},l_{k,2}^{\beta})$: a confirmed pair of conflicting subdivided segments between AGV$\alpha$ and higher-priority AGV$\beta$

The conflict pairs in $\mathcal{C}_{2}^{\alpha}(L_{j}^{\alpha})$ are ordered chronologically by the subdivided segments of AGV$\alpha$. If one subdivided segment conflicts with multiple higher-priority AGVs, they are ordered by the arrival times of the higher-priority AGVs, with earlier arrivals listed first. Simultaneous arrivals do not occur, because once a preceding AGV successfully applies a deconfliction strategy, the arrival times become distinct.

If the precise conflict-information set $\mathcal{C}_{2}^{\alpha}(L_{j}^{\alpha})$ is empty, coarse screening continues on the next variable-length segment $L_{j+1}^{\alpha}$. If the entire path of AGV$\alpha$ has no spatiotemporal conflicts with higher-priority AGVs, no strategy adjustment is needed; retain $\mathcal{D}_{0}^{\alpha}$ and $\mathcal{D}_{1}^{\alpha}$, and store them into the global sets $\mathcal{D}_{0}^{\alpha}$ and $\mathcal{D}_{1}^{\alpha}$, respectively, before proceeding to conflict checking for the next-priority AGV. If $\mathcal{C}_{2}^{\alpha}(L_{j}^{\alpha})$ is nonempty, the process enters the subsequent deconfliction-strategy phase.

**(2) Eliminating AGV path conflicts and deadlocks is an industry challenge. The literature review on AGV path conflicts in section 2.1 is not comprehensive enough and needs to be supplemented. In fact, there are many works studied this issue. For example, [1] and [2] use time-window based search methods to find conflict free paths, [3] and [4] solve conflicts based on area locking, and [5] eliminates path conflicts from the perspective of adjusting AGV task assignments.**

**Response：**

We are very grateful for your additions. After reading the references you provided, we found that our introduction indeed contains citation gaps. We expanded **“Introduction”** to cover **CBS, time-expanded/time-window networks, regional mutual exclusion (area locking)**, and **task-assignment-driven deconfliction**, with the cited works incorporated. We also positioned **GTCD** (priority-based, edge-level checks) for clear contrast. The revised portion of the introduction is as follows:

“The research focus of this paper is on centralized system planning methods. Many centralized system solutions are currently popular, the Conflict Based Search (CBS) achieves collision-free planning via a two-level decomposition: at the lower level, optimal single-agent paths are computed independently without constraints from other agents; at the upper level, upon detecting spatiotemporal occupancy at the vertex or edge level, the corresponding spatiotemporal constraints are introduced and re-planning is triggered, thereby progressively eliminating conflicts [7-10]. CBS can find a globally optimal solution that minimizes the total path cost of all AGVs; however, its drawbacks are evident: because it must compare vertex and edge conflicts across agents, the upper-level branching on constraints and the lower-level re-planning grow increasingly expensive as the number of AGVs and the environmental complexity increase. In parallel to CBS, a line of work based on spatiotemporal networks/time-window search explicitly discretizes time and builds a time-expanded graph, internalizing collision-avoidance constraints into the network via capacity limits and forbidden edges, and then solves the problem using shortest-path or minimum-cost flow methods. This approach is highly verifiable but suffers from rapid state-space growth when the time granularity is fine, the task horizon is long, or the fleet size is large [11-12].Another strand emphasizes regional mutual exclusion: by imposing exclusive occupancy or reservation on bottleneck corridors and intersection areas to preclude passing encounters and concurrent conflicts, it offers a simple engineering implementation; its limitation is that poorly chosen interlock-region granularity induces a trade-off between conservatism and throughput [13-14]. In addition, some studies move conflict suppression upstream to the task-assignment stage by incorporating potential congestion or crossing risks into the cost function and constraints, yielding task–path matches with fewer anticipated conflicts before execution; the drawback is that performance depends on the fidelity of the risk characterization, and local geometric conflicts during operation typically still require online compensation [15].

Beyond the foregoing mature frameworks, another common remedy is to assign AGV priorities and confirm conflicts via a Grid-based Time-window Conflict Detection (GTCD) algorithm. Unlike CBS, GTCD emphasizes priority-based pre-screening: conflicts are detected directly by testing for spatiotemporal overlap on edges, without decomposing into vertex versus edge conflicts or constructing a constraint tree. Any overlap is deemed a conflict and is resolved at runtime through waiting or path re-planning. GTCD is often combined with heuristic path planners such as Particle Swarm Optimization (PSO) [16-17], Genetic Algorithm (GA) [18-19] and Ant Colony Optimization (ACO) [20-21].”

Among these, references 11-15 are precisely the works you recommended.

In addition, at the end of the introduction we have outlined the structure of our paper and the proposed contributions to help readers better grasp our ideas, as follows:

“Overall, this study develops along the CBS framework, adopting a two-level decomposition of “first compute single-agent optima, then perform global deconfliction”: the lower level employs IACO, while the upper level implements deconfliction by combining priority assignments with an RLBCD mechanism. The contributions are summarized as follows:

(i) Propose IACO to improve the path-planning quality of individual AGVs.

(ii) Leverage the priority scheme to map high-priority AGV paths into dynamic-obstacle regions for low-priority AGVs, tightly coupling IACO so that, through the pheromone-positive-feedback mechanism of ant colonies, optimal alternative paths under collision-free constraints are found automatically.

(iii) Introduce an RLBCD mechanism for rapid conflict detection across multiple AGV paths, enhancing detection efficiency while maintaining accuracy.”

**(3) The paper presents an AGV obstacle avoidance strategy that uses waiting and replanning. However, after employing these two methods, conflicts may occur again. How do you address this issue?**

**Response:**

Thank you very much for your detailed question. Your insight is excellent. Indeed, for certain algorithms such as CBS, new conflicts may arise even after applying waiting or restarting, necessitating further conflict detection to handle the newly emerging cases.

In our framework, priorities allow us to pre-assign responsibility for deconfliction: high-priority AGVs have the right of way and do not need to consider conflicts with lower-priority AGVs. Accordingly, we map the subdivided path segments of high-priority AGVs into dynamic-obstacle regions for lower-priority AGVs. The nodes of these regions are dynamically inserted into the tabu list of the ant colony algorithm. When the colony approaches such a region, if the time of moving to the next node overlaps with the activation time window of the dynamic obstacle, that node is placed on the tabu list and cannot be selected. Combined with the pheromone positive-feedback mechanism, once the colony successfully finds a path, the new path is guaranteed to be free of conflicts with higher-priority AGVs. By iterating this process, all AGVs can operate stably without collisions.

For the waiting strategy, it is true that the planned path must be rechecked; however, because priorities are in place, a lower-priority AGV only needs to rerun RLBCD, which quickly identifies the next set of segments that conflict with higher-priority AGVs and thus enables rapid waiting decisions.

We also considered the extreme case you mentioned and added a supplementary safeguard: if the ant colony algorithm still fails within the allowed number of replanning attempts—i.e., at some time instant no feasible solution can be found due to dense dynamic obstacles—and the waiting strategy exceeds its maximum time limit, the current AGV’s task is canceled and deferred to the next departure batch. This measure ensures that already planned high-priority paths remain unaffected. We have incorporated this point in both the introduction and the strategy-selection section of the paper.

“In extreme cases where no deconfliction strategy is feasible for a low-priority AGV at a given time, the algorithm applies a preemptive intervention: the task will be canceled at the current time instant and restarted in the planning for AGVs departing in the next time batch. This strategy is consistent with priority-ordered planning and serves as a feasibility prerequisite for collision-free multi-AGV operation.”

**(4) You mentioned the use of CBS and GTCD methods in your benchmark. However, you did not give a clear explanation of how these two methods were specifically utilized in your study. To enhance the clarity and reproducibility of your research, it would be beneficial for you to briefly introduce the application of CBS and GTCD in your paper.**

**Response:**

Thank you for emphasizing reproducibility—we agree that baseline configurations should be transparent. In **“Experimental Results and Analysis”**, we added a concise paragraph: three methods share the same (x, y, t) inputs and the same conflict criterion (distance below$D_{\text{safe}}$and linear time-window overlap). GTCD applies our priority rule then performs direct edge-level checks; RLBCD encapsulates into co-directional segments with coarse-to-fine screening; CBS uses point/edge checks without priority. The detection count is defined as one full spatiotemporal check per path. We have also provided a more detailed description of GTCD and CBS in the **“Introduction”**; the specific revisions are as follows:

“the Conflict Based Search (CBS) achieves collision-free planning via a two-level decomposition: at the lower level, optimal single-agent paths are computed independently without constraints from other agents; at the upper level, upon detecting spatiotemporal occupancy at the vertex or edge level, the corresponding spatiotemporal constraints are introduced and re-planning is triggered, thereby progressively eliminating conflicts [7-10].”

“Beyond the foregoing mature frameworks, another common remedy is to assign AGV priorities and confirm conflicts via a Grid-based Time-window Conflict Detection (GTCD) algorithm. Unlike CBS, GTCD emphasizes priority-based pre-screening: conflicts are detected directly by testing for spatiotemporal overlap on edges, without decomposing into vertex versus edge conflicts or constructing a constraint tree. Any overlap is deemed a conflict and is resolved at runtime through waiting or path re-planning. GTCD is often combined with heuristic path planners such as Particle Swarm Optimization (PSO) [16-17], Genetic Algorithm (GA) [18-19] and Ant Colony Optimization (ACO) [20-21].”

“Then the RLBCD mechanism is tested centrally, compared with GTCD and CBS conflict detection methods. In these experiments, all three methods are given the same initial spatiotemporal path-node data for the AGVs (**Fig 6**(a)-(f)), obtained by running IACO from the start/end points in **Table 3**. The concrete implementations are as follows: GTCD adopts the priority scheme proposed in this paper to rank AGVs and then performs direct edge-level spatiotemporal conflict checks (using the shortest-edge criterion). RLBCD further processes the path-node data into same-direction polylines and applies a coarse-to-fine spatiotemporal conflict detection. CBS does not assign priorities; it directly performs minimum-edge and minimum-vertex spatiotemporal conflict checks on each AGV’s path-node data. All three methods use a consistent conflict criterion: if the spatial separation is below $D_{\text{safe}}$ and the time windows overlap under linear matching, a conflict is declared. The detection count is recorded once per complete spatiotemporal check of a path, with the objective of locating each AGV’s first conflict position. Finally, we compute the number of path-information checks and the task completion time for each method to validate the proposed approach.”

**(5) After adopting the proposed method, is the conflict rate of AGVs zero for experimental cases of different scales? If not, there should be experiments to demonstrate the improvement of the proposed method in terms of the conflict rate.**

**Response:**

We appreciate the request for scale-sensitivity evidence. To further verify, at the experimental scales considered (3-7 AGVs), whether the algorithm enables collision-free simulation across different fleet sizes, we added supplementary experiments. In particular, we include the actual simulation snapshots and the corresponding Gantt chart for the 7-AGV case to demonstrate the absence of conflicts (**Fig10.**), and we summarize the results for each fleet size in **Table 6**. These additions further clarify, under identical experimental scales, parameter settings, and attempt counts, the differences and relative merits between our proposed algorithm and a NACO-based ant-colony deconfliction strategy. To enable more rigorous data validation, we will provide, in addition to the data reported in the paper, detailed path-node records for all experiments, including computed path lengths, cumulative turning angles, and per-AGV execution details before and after deconfliction following plan acceptance. The newly added experimental section and table are as follows:

**(a)**NACO	**(b)**IACO

**(c)**NACO

**(d)**IACO

**Fig10.** 7 AGVs started at the same time with different algorithms to plan

**Table 6** 3-7 AGVs route planning post-processing

| Indicators | NACO/ IACO | | | | NACO- IACO |  |
| --- | --- | --- | --- | --- | --- | --- |
|  | Path length (m) | Total time(s) | Total turn angle(°) | Conflict | m/s/° |  |
| 3 AGV | 93.77/95.28 | 272.88/250.57 | 1440/1080 | 0/0 | -1.51/22.31/360 | |
| 4 AGV | 118.67/120.43 | 339.84/328.35 | 1845/1575 | 0/0 | -1.79/11.49/270 | |
| 5 AGV | 144.64/148.15 | 422.78/418.81 | 2385/2205 | 0/0 | -3.51/3.97/180 | |
| 6 AGV | 169.88/172.23 | 502.92/491.95 | 2835/2610 | 0/0 | -2.35/10.97/225 | |
| 7 AGV | 199.61/202.30 | 595.71/566.12 | 3330/2880 | 0/0 | -2.69/29.59/450 | |

Note: Values are rounded to two decimal places. NACO (original) uses a fixed 1 s/turn penalty. For cross-method comparability, the corrected NACO total time is recomputed using the same angle-proportional turning loss as IACO, i.e., 10/180 s/°. In addition to turning and path-length costs, the total time also includes waiting-time losses.

References:

[1] Fan, Z., Gu, C., Yin, X., Liu, C., & Huang, H. (2017, December). Time window based path planning of multi-AGVs in logistics center. In 2017 10th International Symposium on Computational Intelligence and Design (ISCID) (Vol. 2, pp. 161-166). IEEE.

[2] Xin, J., Meng, C., Schulte, F., Peng, J., Liu, Y., & Negenborn, R. R. (2020). A time-space network model for collision-free routing of planar motions in a multirobot station. IEEE Transactions on Industrial Informatics, 16(10), 6413-6422.

[3] Kim, K. H., Jeon, S. M., & Ryu, K. R. (2007). Deadlock prevention for automated guided vehicles in automated container terminals. Container Terminals and Cargo Systems: Design, Operations Management, and Logistics Control Issues, 243-263.

[4] Małopolski, W. (2018). A sustainable and conflict-free operation of AGVs in a square topology. Computers & Industrial Engineering, 126, 472-481.

[5] Li, S., Fan, L., & Jia, S. (2024). A hierarchical solution framework for dynamic and conflict-free AGV scheduling in an automated container terminal. Transportation Research Part C: Emerging Technologies, 165, 104724.

**Reviewer #2: After reviewing your manuscript on multi-AGV path planning with RLBCD and IACO, I find the work addresses an important problem in textile industry automation and presents interesting ideas for improving conflict detection efficiency through R-tree indexing and refined ant colony optimization. However, I have identified several issues requiring major revision:**

**1. Missing Theoretical Complexity Analysis**

**The paper lacks theoretical complexity analysis for the RLBCD algorithm. While experimental runtime comparisons are provided, there's no Big-O notation or formal analysis of how the algorithm scales with increasing numbers of AGVs, path lengths, or map sizes. For a paper proposing a new algorithmic approach, this theoretical foundation is essential for readers to understand the fundamental scaling properties beyond your specific implementation and test cases.**

**Response:**

We are deeply honored by your recognition of the innovations in our work. Your positive assessment is both encouraging and motivating. Thank you for insisting on theoretical scalability—this is essential for an algorithmic paper. We added a dedicated subsection, “RLBCD time-complexity,” presenting Big-O bounds for preparation, coarse screening, fine screening, per-segment, and per-AGV costs (Eqs. (20)-(25)), and analyzing the worst-case scenario.

RLBCD time-complexity

In our analysis, no spatial index structure is instantiated; the algorithm operates on the segment–time-window representation directly. We assume constant-time segment-to-segment distance in 2-D and linear matching of time windows, on which the following big-O bounds are derived. Since RLBCD requires the same-direction segment information of higher-priority AGVs for coarse screening, let $N^{\alpha ll}$ denote the total number of nodes belonging to all known higher-priority AGVs together with the current-batch AGVs. The time complexity of the preparation stage is therefore

$$T_{\text{prepare}}\left( \mathcal{D}_{1} \right)=O(N^{\alpha ll})$$

(20)

Assume the total number of segments in the set $\mathcal{D}_{1}$ is $H$. For the AGV$\alpha$ currently under inspection, the number of same-direction segments is $N_{\text{turns}}^{\alpha}+1$. Let the segment being checked be $L_{j}^{\alpha}$, whose number of subdivided segments is $g_{j}^{\alpha}$. After coarse screening by time-window matching and exact distance filtering, the candidate set $\mathcal{C}_{1}^{\alpha}(L_{j}^{\alpha})$ contains $G_{j}$ elements. Hence, the coarse-stage complexity for$L_{j}^{\alpha}$ depends on the total number $H$ of higher-priority segments, giving

$$T_{\text{coarse}}(L_{j}^{\alpha})=O(H)$$

(21)

Entering the fine-screening stage, suppose $G_{j}$ is further subdivided into $K_{j}$elementary segments. Using time-window overlap and exact distance checks, the final number of conflicting subdivided-segment pairs is $Q_{j}$. The fine-stage complexity is

$$T_{\text{fine}}(L_{j}^{\alpha})=O(K_{j}+g_{j}^{\alpha}+(g_{j}^{\alpha}K_{j}))=O(g_{j}^{\alpha}K_{j})$$

(22)

- $K_{j}$, $g_{j}^{\alpha}$：subdivision of $G_{j}$ and $L_{j}^{\alpha}$, each costing $O(1)$ per segment

- $(g_{j}^{\alpha}K_{j})$：pairwise time-window checks between subdivided segments, proportional to the number of checks

Therefore, for the segment $L_{j}^{\alpha}$, the time complexity of one RLBCD execution is

$$T_{\text{RLBCD}}(L_{j}^{\alpha})=O(H+g_{j}^{\alpha}K_{j})$$

(23)

Overall, the time complexity of path conflict detection for AGV$\alpha$ is

$$T_{\text{RLBCD}}(\mathrm{AGV}\alpha)=O({(N}_{\text{turns}}^{\alpha}+1)H+\sum_{j=1}^{N_{\text{turns}}^{\alpha}+1} g_{j}^{\alpha}K_{j})$$

(24)

In the coarse-screening stage, if the time-window and distance checks exhibit heavy overlap, then $G_{j}=H$.

In the fine-screening stage, let the upper bound on the number of subdivisions of any same-direction segment be $g_{max}$. Then $K_{j}\leq Hg_{max}$ and $g_{j}^{\alpha}\leq g_{max}$. The worst case is therefore:

$$T_{\text{coarse}}(L_{j}^{\alpha})=O(H)$$

$$T_{\text{fine}}\left( L_{j}^{\alpha} \right)=O\left( g_{j}^{\alpha}K_{j} \right)\leq O\left( H{g_{max}}^{2} \right)$$

$$T_{\text{RLBCD}}\left( L_{j}^{\alpha} \right)=O\left( H \right)+O\left( H{g_{max}}^{2} \right)=O\left( H{g_{max}}^{2} \right)$$

$$T_{\text{RLBCD}}(\mathrm{AGV}\alpha)=O\left( {(N}_{\text{turns}}^{\alpha}+1)H{g_{max}}^{2} \right)$$

(25)

**2. Inconsistent Time Calculations in Results (Tables 5 & 6)**

**There's a problem with the reported times that calls into question the validity of your experimental comparison. In Table 5, IACO shows identical ideal and actual times (82.6s), yet reports 360° of turning. Based on your equations 4-6 and stated parameters (t_turn_unit = 0.055s), this turning should add 19.8 seconds. With a 31.3m path at 0.5 m/s, the movement time alone is 62.6s, so actual time should be approximately 82.4s, not the same as ideal.**

**This pattern repeats in Table 6 where total ideal equals total actual (250.5s) despite 1080° of cumulative turning across three AGVs. Additionally, NACO's ideal time (70.3s) doesn't match what should be calculated from its path length (60.2s for 30.1m).**

**These inconsistencies suggest either the "ideal time" is defined differently for different algorithms, there are calculation errors, or the turning time model isn't being applied correctly. This needs to be clarified and corrected for meaningful algorithm comparison.**

**Response:**

Thank you for your insightful comments; you are correct that our calculations contained errors. Upon careful verification, we identified three sources. First, path lengths were manually truncated to one decimal place (e.g., 30.1 m instead of 30.14 m), which led the code to round the computed time, e.g., $30.14\times2=60.28\approx60.3$s. Second, for the IACO unit turning time (0.055), we made a unit-definition error (it should be $s/^{\circ}$) and, in the code, we derived this value from ($10s/180^{\circ}$), i.e., ($0.0555\ldots s/^{\circ}$), but mistakenly stored it as 0.055 without rounding. This yielded $0.055\times180=9.9$s, a $0.1s$ discrepancy, which becomes $0.2s$ at $360^{\circ}$. Third, whether based on NACO or IACO, the multi-AGV deconfliction algorithm includes a waiting strategy. Consequently, the timing results for the multi-AGV experiments cannot be inferred solely by back-calculating from turning time and path length, which led to a misunderstanding. This was our oversight, as the paper did not provide a detailed explanation. Accordingly, we made the following revisions: we now uniformly round all computed quantities to two decimal places and refine the definition of $t_{\text{turn}\text{\_}\text{unit}}^{\mathrm{IACO}}$ to $10/180 s/^{\circ}$. In addition, prompted by your observation, we realized that we had not documented the NACO experimental parameters, which could mislead readers; we therefore added $t_{\text{turn}\text{\_}\text{unit}}^{\mathrm{NACO}}$ to Table 1 and clarified the configuration in experiment (iii). To enable more rigorous data validation, we will provide, in addition to the data reported in the paper, detailed path-node records for all experiments, including computed path lengths, cumulative turning angles, and per-AGV execution details before and after deconfliction following plan acceptance. The consolidated modifications are as follows:

**Table 1.** Algorithm parameterization

| **Parameter** | **Setting** | **Parameter** | **Setting** |
| --- | --- | --- | --- |
| $\alpha$ | 1 | M | 50 |
| $\beta$ | 5 | $D_{\text{safe}}/$Map grid size | 1m |
| $t_{\text{turn}\text{\_}\text{unit}}^{\mathrm{IACO}}$ | 10/180 s/° | $t_{\text{turn}\text{\_}\text{unit}}^{\mathrm{NACO}}$ | 1 s/turn |
| $v$ | 0.5m/s | $Q$ | 30 |
| $\Delta t_{\text{buffer}}$ | 0.2s | Maximum Iterations | 100 |
| ${\Delta t}_{max}$ | 30s | Maximum replanning times | 2 |

(iii) Finally, using the AGV task start and end point data in **Table 3**, the overall algorithm proposed in this paper is compared with the NACO-based multi-AGV collision-free strategy algorithm by setting up a single-AGV versus multi-AGV collision-free path planning test to quantify the task completion time, turning angle, and path planning length to validate the effectiveness of the proposed algorithm. Parameter settings are listed in **Table 1**; in NACO, the only difference from IACO is the unit turning time: $t_{\text{turn}\text{\_}\text{unit}}^{\mathrm{NACO}}$ is count-based, whereas $t_{\text{turn}\text{\_}\text{unit}}^{\mathrm{IACO}}$ is angle-based.”

**Table 5** Path planning post-processing

| **Algorithm** | **Performance indicators** | | | | | |
| --- | --- | --- | --- | --- | --- | --- |
|  | Start | End | Ideal time(s) | Actual time(s) | Turn angle(°) | Path length(m) |
| ACO | (1,1) | (18,20) | 60.28 | 102.81 | 765 | 30.14 |
| NACO | (1,1) | (18,20) | 70.28 | 85.28 | 450 | 30.14 |
| IACO | (1,1) | (18,20) | 82.64 | 82.64 | 360 | 31.31 |

Note: Values are rounded to two decimal places. For ACO and NACO, the Ideal time and Actual time are computed as follows:

$$Ideal time\left（ ACO \right）=\frac{Path length}{v},Ideal time\left（ NACO \right）=\frac{Path length}{v}+t_{\text{turn}\text{\_}\text{unit}}^{NACO}\times\text{Turn times }$$

$$\text{ }Actual time(ACO/NACO)=\frac{Path length}{v}+t_{\text{turn}\text{\_}\text{unit}}^{IACO}\times\text{Turn angle}$$

**Table 6** 3-7 AGVs route planning post-processing

| Indicators | NACO/ IACO | | | | NACO- IACO |  |
| --- | --- | --- | --- | --- | --- | --- |
|  | Path length (m) | Total time(s) | Total turn angle(°) | Conflict | m/s/° |  |
| 3 AGV | 93.77/95.28 | 272.88/250.57 | 1440/1080 | 0/0 | -1.51/22.31/360 | |
| 4 AGV | 118.67/120.43 | 339.84/328.35 | 1845/1575 | 0/0 | -1.79/11.49/270 | |
| 5 AGV | 144.64/148.15 | 422.78/418.81 | 2385/2205 | 0/0 | -3.51/3.97/180 | |
| 6 AGV | 169.88/172.23 | 502.92/491.95 | 2835/2610 | 0/0 | -2.35/10.97/225 | |
| 7 AGV | 199.61/202.30 | 595.71/566.12 | 3330/2880 | 0/0 | -2.69/29.59/450 | |

Note: Values are rounded to two decimal places. NACO (original) uses a fixed 1 s/turn penalty. For cross-method comparability, the corrected NACO total time is recomputed using the same angle-proportional turning loss as IACO, i.e., 10/180 s/°. In addition to turning and path-length costs, the total time also includes waiting-time losses.

**3. Minor Syntax Issue**

**Algorithm 1, line 6: "determine θᵢ in using relative direction of neighboring three nodes" - the word "in" appears to be either a typo or incomplete phrase. Should read "determine θᵢ using relative direction..."**

**Response:**

Thank you very much for pointing out the grammatical errors; we have made the corresponding revisions.

We sincerely appreciate the reviewers’ deep insights, which prompted us to unify the timing model and rounding, clarify baseline configurations, strengthen theoretical analysis, and make the deconfliction logic more explicit. Thank you again for your time and consideration.
